# Testing the Effect of Mountain Ranges as a Physical Barrier to Current Gene Flow and Environmentally Dependent Adaptive Divergence in *Cunninghamia konishii* (Cupressaceae)

**DOI:** 10.3389/fgene.2019.00742

**Published:** 2019-08-09

**Authors:** Yi-Shao Li, Kai-Ming Shih, Chung-Te Chang, Jeng-Der Chung, Shih-Ying Hwang

**Affiliations:** ^1^School of Life Science, National Taiwan Normal University, Taipei, Taiwan; ^2^Department of Life Science, Tunghai University, Taichung, Taiwan; ^3^Division of Silviculture, Taiwan Forestry Research Institute, Taipei, Taiwan

**Keywords:** AFLP, barriers to gene flow, conservation, Cunninghamia konishii, mountain ranges

## Abstract

Populations can be genetically isolated by differences in their ecology or environment that hampered efficient migration, or they may be isolated solely by geographic distance. Moreover, mountain ranges across a species’ distribution area might have acted as barriers to gene flow. Genetic variation was quantified using amplified fragment length polymorphism (AFLP) and 13 selective amplification primer combinations used generated a total of 482 fragments. Here, we tested the barrier effects of mountains on gene flow and environmentally dependent local adaptation of *Cunninghamia konishii* occur in Taiwan. A pattern of genetic isolation by distance was not found and variation partitioning revealed that environment explained a relatively larger proportion of genetic variation than geography. The effect of mountains as barriers to genetic exchange, despite low population differentiation indicating a high rate of gene flow, was found within the distribution range of *C. konishii*. Twelve AFLP loci were identified as potential selective outliers using genome-scan methods (BAYESCAN and DFDIST) and strongly associated with environmental variables using regression approaches (LFMM, Samβada, and rstanarm) demonstrating adaptive divergence underlying local adaptation. Annual mean temperature, annual precipitation, and slope could be the most important environmental factors causally associated with adaptive genetic variation in *C. konishii*. The study revealed the existence of physical barriers to current gene flow and environmentally dependent adaptive divergence, and a significant proportion of the rate of gene flow may represent a reflection of demographic history.

## Introduction

Understanding connectivity among populations and pattern of gene flow has been important for deciphering how species adapt in response to environments and has clear practical implications for conservation of forest genetic resources (Allendorf et al., 2010; Aitken and Bemmels, 2016). Low levels of population genetic differentiation are commonly found in coniferous species due to efficient gene flow based on paternally inherited plastid DNA (ptDNA) variation (Neale et al., 1991; Hipkins et al., 1994; Hwang et al., 2003) and biparentally inherited molecular markers, such as allozyme (Hamrick et al., 1992; Lin et al., 1993; Hamrick and Godt, 1996; Lin et al., 1998). Although gene flow maintains genetic diversity and is critical to population resilience and persistence (Allendorf et al., 2013), high rate of gene flow among closely related sexual populations can preclude local adaptation by eroding divergence driven by natural selection due to the homogenizing effect of dispersing individuals mating among populations (García-Ramos and Kirkpatrick, 1997), resulting in no population adaptive divergence (Li et al., 2018). Nevertheless, selection invoked by environmental heterogeneity can counteract the effect of gene flow (Lenormand, 2002). The failure of detecting variation under selection might have also related to past demographic histories and stochastic mechanisms (Lande, 1988; Wang et al., 2017). It is likely that the balance between migration and selection is species dependent and is related to the realized ecological niche where species can tolerate in response to ecological factors (Pulliam, 2000; Bruno et al., 2003).

The accumulation of genetic variation can provide as raw materials for evolutionary potential under natural selection (Petit and Hampe, 2006; Barrett and Schluter, 2008). The investigation involved orthologous coding sequences in gymnosperms and angiosperms showed that the average synonymous mutation rate was found to be higher in angiosperms in contrast to gymnosperms (Buschiazzo et al., 2012). However, the rate of nonsynonymous substitutions in protein-coding genes was found to be higher in conifers compared with angiosperms suggesting that conifers harbored higher number of fixed adaptive mutations than angiosperms (Buschiazzo et al., 2012), and it is common to observe population local adaptation of conifers in response to environmental heterogeneity (e.g., Mimura and Aitken, 2010; Grivet et al., 2011; Chen et al., 2012; Fang et al., 2013; Shih et al., 2018). However, no adaptive genetic divergence between populations of *Taiwania cryptomerioides* occurring in Taiwan was detected probably due to random neutral drift because of the high rate of gene flow among populations that eroded the adaptive local mutations (Li et al., 2018).

Natural selection driven by ecological factors will result in the development of ecological adaptation and divergence, and selection can act on genetic variation (e.g., Holderegger et al., 2008; Chen et al., 2012; Fang et al., 2013). Genetic variation in natural populations of a species can be quantified using methods involving next-generation sequencing (NGS), such as restriction site-associated sequencing (Shih et al., 2018). Although less powerful than NGS technologies, amplified fragment length polymorphism (AFLP) is an efficient approach allows generation of hundreds of molecular markers from genome sequences of nonmodel organisms to identify candidate genetic variation involved in adaptive evolution that derived from DNA sequence variation (Holderegger et al., 2008; Chen et al., 2012; Fang et al., 2013).

**Figure 1:**
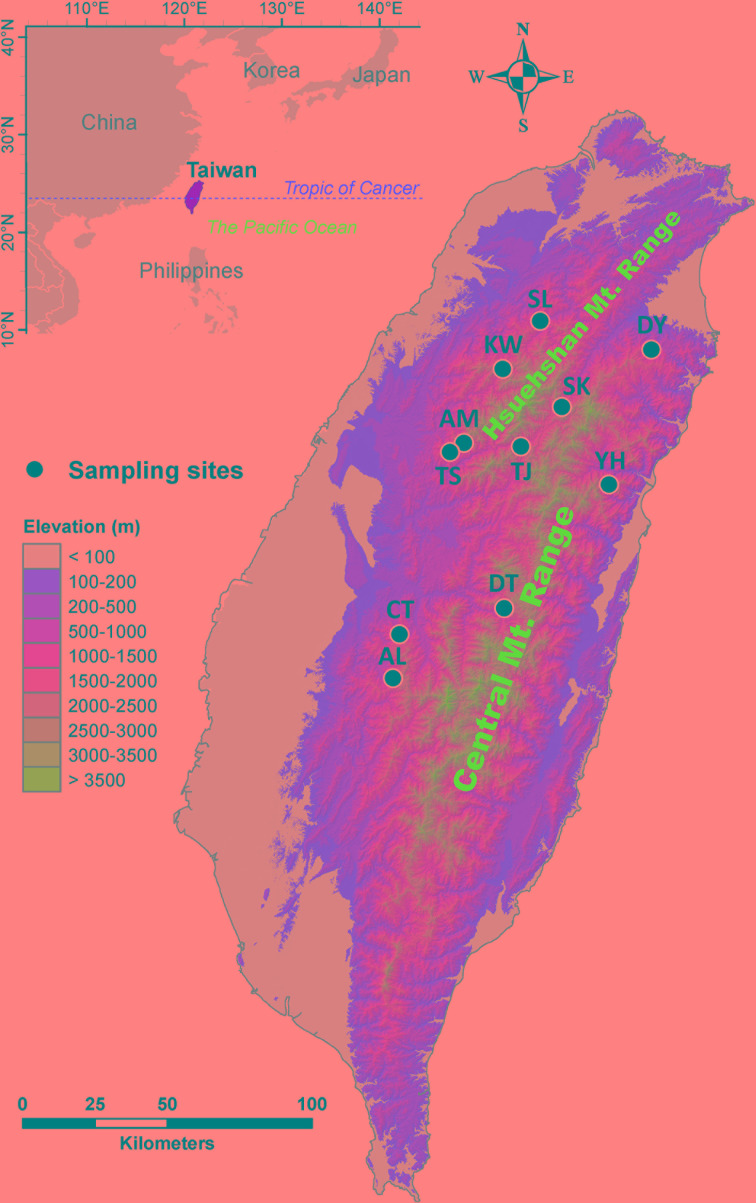
Geographic distribution of the 11 populations of *C. konishii* occur in Taiwan. See **Table 1** for abbreviations of the 11 populations of *C. konishii*.

*Cunninghamia konishii* Hayata (Cupressaceae) is a coniferous species disjunctly distributed in northern and central Taiwan and along part of the border between Vietnam and Lao People’s Democratic Republic (Nga et al., 2016; http://threatenedconifers.rbge.org.uk/taxa/details/cunninghamia-konishii). *C. konishii* is closely related to *Cunninghamia lanceolata* (Lamb). Hook. distributed in mainland China (Lin et al., 1998; Chung et al., 2004) and it may represent a montane form rather than a distinct species (http://threatenedconifers.rbge.org.uk/taxa/details/cunninghamia-konishii). In Taiwan, *C. konishii* is a dominant species among other conifers associated mainly with *Chamaecyparis*, *Pinus* spp., and *Pseudotsuga wilsoniana* at elevations of 1,300–2,800 m (Liu, 1966). Human interferences have greatly impacted this species, e.g., a pure stand of *C. konishii*(ca. 60 hectares) in Shiangshanshan, Hsinchu County, northwest Taiwan, was entirely vanished by logging. Low level of population differentiation was revealed for *C. konishii* based on allozyme (Lin et al., 1998) and ptDNA (Hwang et al., 2003). However, Chung et al. (2004) found high level of genetic differentiation among *C. konishii* populations based on 357 AFLP markers derived from three AFLP selective amplification primer pairs. Populations of *C. konishii* are separated not only by major mountain ranges including the Hsuehshan Mountain Range (HMR) and the Central Mountain Range (CMR) trending from north to south, but also by mountains in between of the HMR and the CMR (**Figure 1**). Mountains were revealed to be the third important factor, after tectonics and climate, as a physical barrier to dispersal in the biogeographic differentiation of a species (Antonelli, 2017). It is likely that mountains may have had an active role in population dispersal of *C. konishii*. In addition, the relationship between genetic variation in *C. konishii* and environmental variables implying local adaptation has not been tested. Investigation assesses the association of genetic variation with environment is important for the conservation program because identification of environmentally dependent ecotypes can be crucial in the assisted migration program of *C. konishii*, particularly in the face of global climate change (Aitken et al., 2008; Aitken and Whitlock, 2013; Aitken and Bemmels, 2016).

Environment may play an important role in driving population divergence (Wang et al., 2013; Sexton et al., 2014). However, population differentiation may simply reflect a correlation between genetic and geographic distance independent of environmental conditions (Hutchison and Templeton, 1999). *C. konishii* populations occupy a wide elevational band (∼1,500 m), though within a small latitudinal range, with wide variety of habitats because of varied geographical topologies and climates (Su, 1984; Li et al., 2013). It is likely that mountains and environment both have been critically involved in shaping the population structure of *C. konishii*. Therefore, genetic variation of 115 individuals from 11 populations was surveyed using 13 AFLP selective primer pairs. Genetic variation and sample site coordinates were used in testing the hypothesis of mountain ranges as a physical dispersal barrier to genetic exchange and genetic variation together with information of environmental variables were used to test environmentally dependent adaptive divergence underlying local adaptation. The specific aims of the present study were to 1) test the barrier effects of mountains to current gene flow and 2) assess the relationships of genetic variation with environmental variables underlying local adaptation, in the light of usefulness in the future conservation of *C. konishii*.

## Materials and Methods

### Sampling and Genotyping

We collected 115 individuals of *C. konishii* from 11 populations (**Table 1**, **Figure 1**). Leaf samples dried with silica gel were used for DNA extraction (Dehestani and Kazemi Tabar, 2007). We quantified genetic variation using AFLP (Vos et al., 1995). In selective amplification, 13 *Eco*RI-*Mse*I primer combinations (E00: 5’-GACTGCGTACCAATTC-3’ and M00: 5’-GATGAGTCCTGAGTAA-3’) with additional five and three bases were added, respectively, at the ends, were used (**Supplementary Table 1**). Fragments amplified by polymerase chain reaction were electrophoresed on an ABI 3730XL DNA analyzer (Applied Biosystem, Foster City, CA, USA) and scored with Peak Scanner v.1.0 (Applied Biosystem). The presence and absence of amplified fragments were scored with the fluorescent threshold set at 150 units. Amplified fragment peaks in the range of 150–500 base pair (bp) separated by less than one nucleotide in a ± 0.8 bp window were scored as the same fragment. Markers scored higher than 99% or less than 1% of individuals were removed. The AFLP dataset was deposited in **Supplementary Data Sheet 1**.

**Table 1:** Site properties and population genetic parameters based on 482 amplified fragment length polymorphism (AFLP) loci of the sampled *C. konishii* populations.

Locality	Latitude/Longitude	Altitude (m)	*N*	*PBr*	*%P*	*uH*_E_ (SE)	*I*_A_ (*P*)	*r*D (*P*)
Alishan (AL)	120.7644/23.5303	2,132	19	0.0261	73.7	0.260 (0.008)	0.923 (0.001)	0.003 (0.001)
Anmashan (AM)	121.0044/24.2647	2,510	6	0.0110	41.1	0.239 (0.008)	−0.076 (0.519)	−0.0004 (0.519)
Chitou (CT)	120.7864/23.6675	1,236	3	0.0225	33.0	0.284 (0.008)	0.491 (0.241)	0.003 (0.241)
Denta (DT)	121.1408/23.7489	2,299	22	0.0119	67.4	0.227 (0.008)	2.010 (0.001)	0.006 (0.001)
Dayuanshan (DY)	121.6436/24.5544	1,065	3	0.0296	32.2	0.262 (0.009)	5.123 (0.003)	0.033 (0.003)
Kuanwu (KW)	121.1375/24.4956	2,060	19	0.0149	74.3	0.253 (0.008)	3.213 (0.001)	0.009 (0.001)
Shengkuang (SK)	121.3381/24.3772	2,176	9	0.0141	60.8	0.265 (0.009)	4.798 (0.001)	0.017 (0.001)
Shiouhluan (SL)	121.2642/24.6458	1,296	10	0.0188	58.9	0.251 (0.009)	0.046 (0.409)	0.0002 (0.409)
Tajian (TJ)	121.1989/24.2542	1,511	10	0.0125	58.1	0.243 (0.009)	1.811 (0.001)	0.007 (0.001)
Tashueshan (TS)	120.9569/24.2358	1,810	8	0.0157	49.4	0.239 (0.009)	2.493 (0.001)	0.011 (0.001)
Yeinhai (YH)	121.4969/24.1336	1,844	6	0.0098	43.6	0.245 (0.009)	0.664 (0.075)	0.003 (0.075)
Total			115					
Average (SD)				0.0170 (0.007)	53.9 (15.1)	0.252 (0.016)		

N, number of samples; uH_E,_ unbiased expected heterozygosity. I_A_, index of association; rD, modified index of association. PBr, private band richness. %P, the percentage of polymorphic loci.

Genotyping error rate of each primer combination was estimated based on the ratio of mismatches in three amplification replicates of three samples in each population. Loci with error rates greater than 5% were removed (Bonin et al., 2004) and the mean error rate was 1.91% (**Supplementary Table 1**).

### Genetic Diversity

The percentage of polymorphic loci (%*P*) at the 95% level with rarefaction to the smallest sample size (*n* = 3) was calculated using AFLPDIV software v.1.1 (Coart et al., 2005). The private band richness (*PBr*) was estimated separately for each population using ADZE software v.1.0 (Szpiech et al., 2008) with rarefaction (Kalinowski, 2004). We calculated index of association (*I*_A_) (Brown et al., 1980) and modified index of association (*r*D) (Agapow and Burt, 2001) using the *ia* function of R poppr package (Kamvar et al., 2014; Kamvar et al., 2015) in the R environment (R Development Core Team, 2018) to assess the level of multilocus linkage disequilibrium (LD), and significant departure from zero was tested with 999 permutations. Unbiased expected heterozygosity (*uH*_E_) (Nei, 1987) within a population were computed using AFLP-SURV v.1.0 (Vekemans et al., 2002). In AFLP-SURV, allele frequencies were estimated assuming Hardy–Weinberg equilibrium with nonuniform prior distribution (Zhivotovsky, 1999) for *uH*_E_ computation. Per locus *uH*_E_ was calculated using ARLEQUIN v.6.0 (Excoffier and Lischer, 2010). Assessment of significant difference of mean *uH*_E_ per locus between populations was performed using a linear mixed effect model (LMM) with population and locus treated as fixed and random effect, respectively, based on reduced maximum likelihood estimation using the *lmer* function of R lme4 package (Bates et al., 2015). Significance was assessed based on type II Wald χ^2^ test using the *Anova* function of R car package (Fox and Weisberg, 2011), and *P* values were adjusted with Tukey’s *post hoc* method using the *lsmeans* function of R emmeans package (Lenth, 2018).

### Genetic Differentiation

The level of genetic differentiation among populations was analyzed *via* analysis of molecular variance (AMOVA) using the *poppr.amova* function of R package poppr, and significance tested using the *randtest* function of R package ade4 with 9,999 permutations (Dray and Dufour, 2007). Across population *F*_ST_ was computed using AFLP-SURV with 9,999 permutations. ARLEQUIN was used to compute pairwise *F*_ST_ with 10,000 permutations. Moreover, HICKORY v.1.1 (Holsinger and Lewis, 2003) was used to estimate θ^II^ (an analog to *F*_ST_), considering the uncertainty associate with the inbreeding coefficient (*f*) for dominant markers. To check the convergence of parameters, two HICKORY runs were performed with sampling and chain length parameters include burnin = 5,000, samples = 100,000, and thinning = 20. Genetic data was evaluated fitting to the full model, *f* = 0 model, θ*^II^* = 0 model, and *f*-free model, and the best fitting model in estimating θ*^II^* was determined by the deviance information criterion (DIC). The *f*-free model was used to estimate *f* within populations because other models produced unreliable *f* estimates. The *f*-free model selects *f* values from a uniform prior distribution without generating a posterior distribution of *f* independent of assumptions.

### Genetic Clustering and Testing the Barrier Effects of Mountains on Gene Flow

Genetic homogeneous groups of *C. konishii* individuals were assessed based on sparse nonnegative factorization (sNMF) algorithm (Frichot and Francois, 2015) and discriminant analysis of principal components (DAPC) (Jombart et al., 2010). Individual assignments based on the least-squares optimization using the *snmf* function of R LEA package (Frichot and Francois, 2015) was performed for *K* = 1–11. The regularization parameter, iterations, and repetitions in *snmf* were set to 100, 200, and 10, respectively, with other arguments set to defaults. The best *K* in LEA was evaluated with the means of minimal cross-entropy (CE). The *find.clusters* and *dapc* functions of R adegenet package (Jombart and Ahmed, 2011) were used in DAPC analysis, in which a principal component analysis (PCA) was first performed and followed by a discriminant analysis that maximize variance between groups. The best *K* in DAPC was indicated by an elbow in the curve of Bayesian information criterion (BIC), as suggested by the authors of the R adegenet package, estimated using the *find.clusters* function.

Given the heterogeneous nature of *C. konishii* populations distributed across mountains, Monmonier’s maximum difference algorithm (Monmonier, 1973; Manni et al., 2004) was used to assess sharp genetic discontinuities in the geographic distribution area of *C. konishii*, based on the Euclidean distance of AFLP and sample coordinates, using the *optimize.monmonier* function of R adegenet package. In *optimize.monmonier*, 10 different starting points were used to find the largest sum of local distances that explains genetic distances among populations.

### Environmental Variables and Heterogeneity

Environmental variables used were 19 bioclimate, 2 topological, and 12 ecological variables. Nineteen bioclimate variables for sample sites at 30-s spatial resolution (∼1 km) were downloaded from the WorldClim v.1.4 (Hijmans et al., 2005). Topographic variables including aspect and slope at 30-m resolution were obtained from Aster Global Digital Elevation Map (https://asterweb.jpl.nasa.gov/gdem.asp). We obtained ecological factors including normalized difference vegetation index (NDVI) and enhanced vegetation index (EVI) derived from moderate resolution imaging spectroradiometer (MODIS) dataset MOD13A2 (1-km resolution), and leaf area index (LAI) and fraction of absorbed photosynthetically active radiation (fPAR) derived from MOD15A2 dataset (500-m resolution). The annual total potential evapotranspiration (PET) was calculated based on MOD16A3 dataset (500-m resolution). All the MODIS datasets were acquired from Land Process Distributed Active Archive Center (http://lpdaac.usgs.gov) during 2001‒2013, and monthly mean values of NDVI, EVI, LAI, and fPAR were computed using a maximum-value composite procedure (Huete et al., 2002). Monthly mean values of the other five ecological factors including relative humidity (RH), cloud cover (CLO), time of sunshine (SunH), number of rainfall days per year (RainD), and mean wind speed (WSmean) were also calculated for data obtained from the Data Bank for Atmospheric & Hydrologic Research (https://dbahr.pccu.edu.tw/, recorded in 1990–2013) at spatial resolution of 1 km using a universal spherical model of the Kriging method in ArcGIS (Chang et al., 2014). Additionally, we acquired soil pH values of sample sites based on an island-wide soil investigation (*n* = 1,150) conducted in 1969‒1986 from the Agriculture and Food Agency of Taiwan (Chang et al., 2009). Annual precipitation and annual PET (derived from annual mean temperature) were used to calculate annual moisture index (Thornthwaite, 1948).

The *cor* function of R was used to calculate correlation coefficients between environmental variables. Variance inflation factor (VIF) was calculated using the *vif* function of R package usdm (Naimi et al., 2014). We retained eight environmental variables for further use based on environmental variables with VIF < 10 and which not strongly correlated with other variables (|*r*| < 0.8). The eight retained environmental variables were aspect, BIO1 (annual mean temperature), BIO7 (annual temperature range), BIO12 (annual precipitation), NDVI, PET, RainD, and slope (**Supplementary Table 2**). Environmental Euclidean distance matrix was used in a permutational multivariate analysis of variance (PERMANOVA) to assess environmental heterogeneity among sample sites and among population genetic clusters (revealed by LEA and DAPC analyses, see *Results*) using the *adonis* function of R package vegan (Oksanen et al., 2017). Pairwise population and cluster comparisons were also performed using the *pairwise.perm.manova* function of R package RVAideMemoire (Herve, 2018). Significant pairwise comparisons were tested with 999 permutations and a false discovery rate (FDR) of 5%.

Mantel test was used to assess genetic isolation by distance (IBD) by analyzing the correlation of the population Euclidean distance matrix of AFLP with the population Euclidean distance matrix of geography (latitude and longitude) using the *mantel* function of R vegan package.

### Partitioning of Genetic Variation Explained by Environmental Variables

The eight retained environmental variables were used in a redundancy analysis (RDA) to assess the relative contribution of environmental variables explaining the total AFLP variation using the *varpart* function of R package vegan, and significance tested using the *anova.cca* function with 999 permutations (Oksanen et al., 2017). The total variation was partitioned into four fractions: pure environmental variables (fraction [a]), geographically structured environmental variables (fraction [b]), pure geographic variables (fraction [c]), and residual effects (fraction [d]) (Borcard et al., 1992; Borcard and Legendre, 2002). Adjusted *R* ^2^ value was used to represent the amount of variation explained in each fraction (Peres-Neto et al., 2006). Longitude and latitude of sample sites were used as geographic variables in the analysis.

### Test for *F*_ST_ Outliers

Two genome scan methods, BAYESCAN and DFDIST, were used to identify *F*_ST_ outliers indicating signature of selection across populations. Hierarchical Bayesian method implemented in BAYESCAN v.2.1 (Foll and Gaggiotti, 2008) uses a reversible-jump Markov chain Monte Carlo algorithm to estimate the ratio of posterior probabilities of selection over neutrality [the posterior odds (PO)]. We used 100 pilot runs of 50,000 iterations followed by a sample size of 50,000 with thinning interval of 20 among 10^6^ iterations in BAYESCAN analysis. A logarithmic scale for model choice of selection over neutrality can be defined as: substantial (log_10_PO > 0.5); strong (log_10_PO > 1.0); very strong (log_10_PO > 1.5); and decisive (log_10_PO > 2) (Jeffreys, 1961; Foll, 2012). We considered a locus with log_10_PO > 0.5 as a potential selective outlier under selection.

DFDIST incorporates the Beaumont and Nichols model modified for AFLP (Beaumont and Nichols, 1996). The probability of a locus that may be under selection by observed *F*_ST_ (Weir and Cockerham, 1984) and *uH*_E_ (Zhivotovsky, 1999) compared to simulated neutral distributions was estimated. Parameters for running DFDIST were: critical frequency = 0.99; Zhivotovsky parameters = 0.25; trimmed mean *F*_ST_ = 0.3 (excluding 30% of highest and 30% of lowest *F*_ST_ values); smoothing proportion = 0.06; 500,000 resamplings; critical *P* = 0.05; and level of differentiation (target average θ) = 0.063254. Empirical loci considered to be the outliers potentially under directional selection were those with *F*_ST_ values significantly greater (*P* < 0.01) than the simulated distribution.

### Test for Associations of Genetic Variation With Environmental Variables

Associations of genetic variation with environmental variables were assessed using Samβada v.0.8.1 (Stucki et al., 2017) and latent factor mixed model (LFMM) (Frichot et al., 2013). A multiple univariate logistic regression approach was employed in Samβada to test for significant correlations of allele frequencies with the values of environmental variables. Models with and without environmental variables were compared, and significant fit was determined based on both Wald and G scores with an FDR cutoff of 0.05. LFMM is a method uses a hierarchical Bayesian mixed model considering background levels of population structure as random effects due to demographic history and IBD patterns, and is introduced *via* latent factors, *K*. In LFMM, genetic data matrix was used as fixed effect in a testing procedure based on *Z*-scores. The number of latent factors, *K*, was set to 3 (based on the LEA and DAPC clustering results). We ran LFMM five times for each value of *K* with 10,000 iterations in Gibbs sampling algorithm and a burn-in period of 5,000 cycles. Z-scores from five independent replicate runs were combined using Fisher–Stouffer method (Brown, 1975), and the resulting *P* values were adjusted using the genomic inflation factor (λ). *P* values were further adjusted based on an FDR correction of 1% using the R qvalue package (Storey et al., 2019).

For those potential selective outliers identified by either genome scan method (BAYESCAN or DFDIST), a Bayesian logistic regression approach implemented in the *stan_glm* function of R rstanarm package (Goodrich et al., 2018) was also used to test for the associations of the potential selective *F*_ST_ outliers with environmental variables. In *stan_glm* analysis, Student’s *t* distribution with mean zero and 7 degrees of freedom was used as the weakly informative prior. The scale of the prior distribution was 10 for the intercept and 2.5 for the predictors. All models were run with four chains for 1,000 warm-up and 1,000 sampling steps. Credible intervals (95% and 99%) of estimate were calculated using the *posterior_interval* function of R rstanarm package.

## Results

### Diversity and Differentiation

We obtained 482 AFLP loci (mean ± SD: 37.08 ± 7.87) for *C. konishii* (**Supplementary Table 1**). The average value of *PBr* ± SD was 0.017 ± 0.007 (ranged from 0.0098 for the population YH to 0.0296 for the population DY) (**Table 1**). The average value of *%P* ± SD was 53.9 ± 15.1 (ranged from 32.2 for the population DY to 74.3 for the population KW). The average level of *uH*_E_ ± SD was 0.252 ± 0.016 and ranged between 0.227 (population DT) and 0.284 (population CT). Within *C. konishii*, the level of *uH*_E_ per locus was significantly different among populations using LMM (χ^2^ = 41.38, *P* < 0.0001). However, significant difference in the level of *uH*_E_ per locus was not found for all pairwise comparisons (**Supplementary Table 3**). No positive correlation of sample size with population *uH*_E_ (*S* = 305.78, ρ = −0.390, *P* = 0.2358) and *PBr* (*S* = 251.29, ρ = −0.142, *P* = 0.6766), respectively, was found based on Spearman’s rank correlation test. However, significant positive correlation was found between sample size and the percentage of polymorphism (*S* = 14.12, ρ = 0.936, *P* < 0.001). Using HICKORY, an estimate of *f* within population was 0.4969 (95% CI: 0.022–0.976) using the *f*-free model representing the contemporary reproductive mode of *C. konishii* (**Supplementary Table 4**). The measures of multilocus LD, *I*_A_ and *r*D, showed significant departure from random association between AFLP loci in 7 (populations AL, DT, DY, KW, SK, TJ, and TS) of the 11 populations examined (**Table 1**).

The *full* model was the best model fitting to the genetic data based on DIC in HICKORY analysis (**Supplementary Table 4**). Low, albeit significant, level of genetic differentiation of *C. konishii* populations was found based on AMOVA and θ^II^ (Φ_ST_ = 0.0827, θ^II^ = 0.0848; **Table 2**, **Supplementary Table 4**). Across population *F*_ST_ estimated using AFLP-SURV was 0.0656 (*P* < 0.001). Significant population pairwise *F*_ST_ estimated using ARLEQUIN was commonly found (**Supplementary Table 5**).

**Table 2:** Summary of the analysis of molecular variance (AMOVA).

Source of variation	df	Sum of squares	Variance component	% variation	Fixation index	*P* value
Between populations	10	1,001.39	4.73	8.27	Φ_ST_ = 0.0827	<0.001
Within populations	104	5,457.78	52.48	91.73		
Total	114	6,459.17	57.21	100		

### Genetic Clustering and Test for IBD

**Figure 2:**
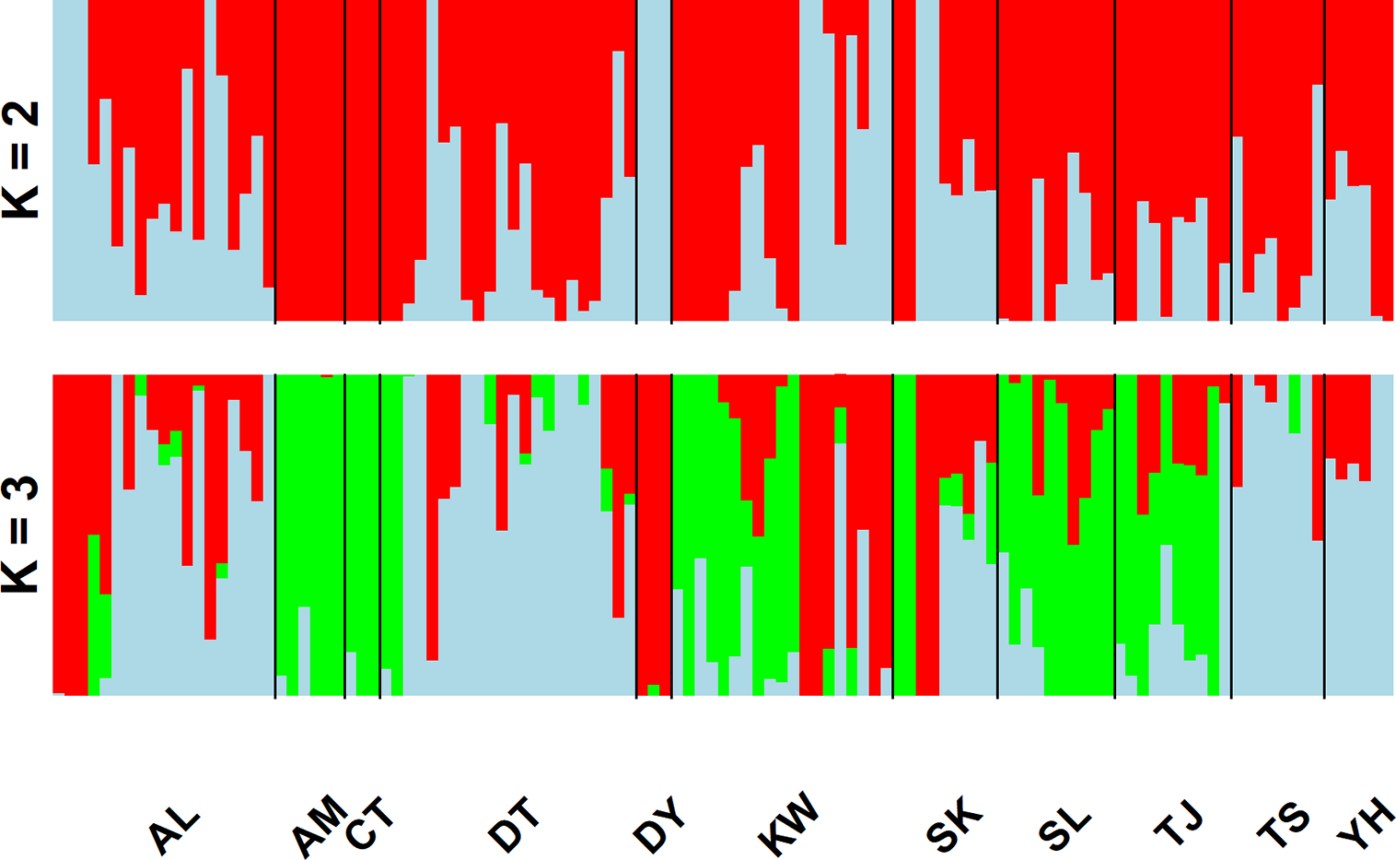
Individual assignments of 115 individuals from 11 populations of *C. konishii* analyzed using LEA. The clustering scenarios for *K* = 2–3 were displayed.

**Figure 3:**
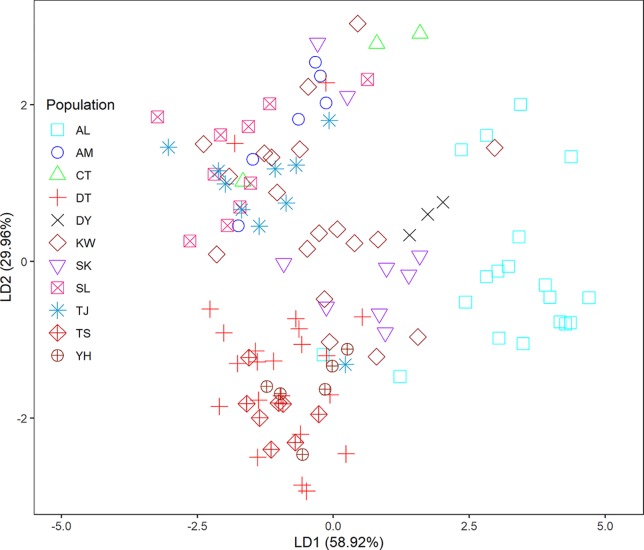
Clustering results analyzed using discriminant analysis of principal components (DAPC).

**Figure 4:**
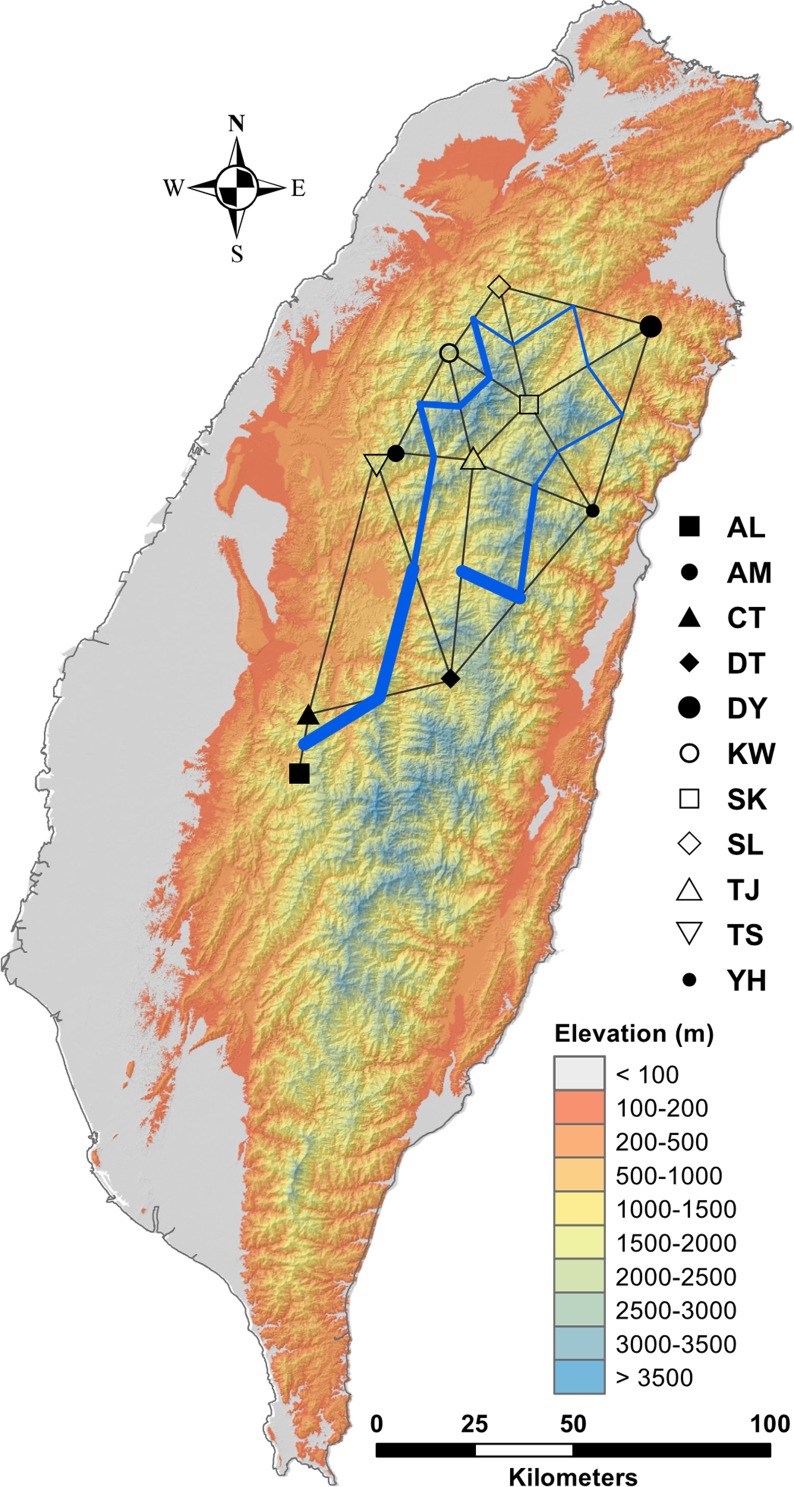
Barriers to genetic exchange identified using Monmnier’s algorithm. The levels of physical barrier effect of gene flow were represented by the thickness of blue lines. See **Table 1** for abbreviations of the 11 populations of *C. konishii*.

In the LEA analysis, the means of minimal CE was minimized at *K* = 3 (**Supplementary Figure 1A**). LEA clustering results were depicted for *K* = 2–3 (**Figure 2**). The lowest BIC was found at *K* = 5, but elbowed at *K* = 3, in DAPC analysis (**Supplementary Figure 1B**). The eigenvalues for the first two retained PCs were 1.04 and 0.64 (**Supplementary Figure 1C**) and the eigenvalues for the first two DAPC linear discriminants were 28.77 and 14.63 (**Supplementary Figure 1D**). Three population clusters can be distinguished based on DAPC: cluster 1 contains population AL; cluster 2 contains populations DT, TS, and YH; and cluster 3 contains populations AM, CT, DY, KW, SK, SL, and TJ. However, a few individuals from one population can be grouped with individuals of other populations in a separate cluster (**Figure 3**). **Figure 4** displayed the spatial boundaries of genetic exchange among *C. konishii* populations using Monmonier’s algorithm. Mantel test revealed no significant genetic isolation by geographic distance (*r*_M_ = 0.251, *P* = 0.107).

### Variation Partitioning of Genetic Variation Explained by Environment and Geography

PERMANOVA revealed significant environmental difference among (*P* = 0.001) and between the three genetic clusters (*P* = 0.001). Significant pairwise differences between populations were found (**Supplementary Table 6**), albeit PERMANOVA revealed no environmental difference across populations (*P* = 1). Total explainable genetic variation was 7.38% (**Table 3**). This value was relatively small as compared to the amounts of unexplained variation (92.62%). Nevertheless, significant amount of genetic variation was explained by both pure environment (5.18%, fraction [a]) and pure geography (1.84%, fraction [c]), albeit small, based on *F* tests of RDA model. Moreover, environment explained a relatively larger proportion of genetic variation than geography.

**Table 3:** The percentage of variation explained in genetic loci accounted for by non-geographically structured environmental variables [a], shared (geographically structured) environmental variables [b], pure geographic factors [c], and undetermined component [d] analyzed based on the eight retained environmental variables.

	Variation (adjusted *R*^2^)	*F*	*P*
Environment [a]	0.05183	1.7834	0.001
Environment + Geography [b]	0.00356	–	–
Geography [c]	0.01840	2.0527	0.001
[a + b + c]	0.07379	1.9082	0.001
Residuals [d]	0.92621	–	–

Proportions of explained variation were obtained from variation partitioning by redundant analysis. F and P values are specified for testable fractions. Fraction [b] is untestable because the adjusted R^2^ value was obtained by subtraction ([a + b + c] − [a] − [c]) rather than by estimation.

### Potential Outliers Strongly Correlated With Environmental Variables

**Figure 5:**
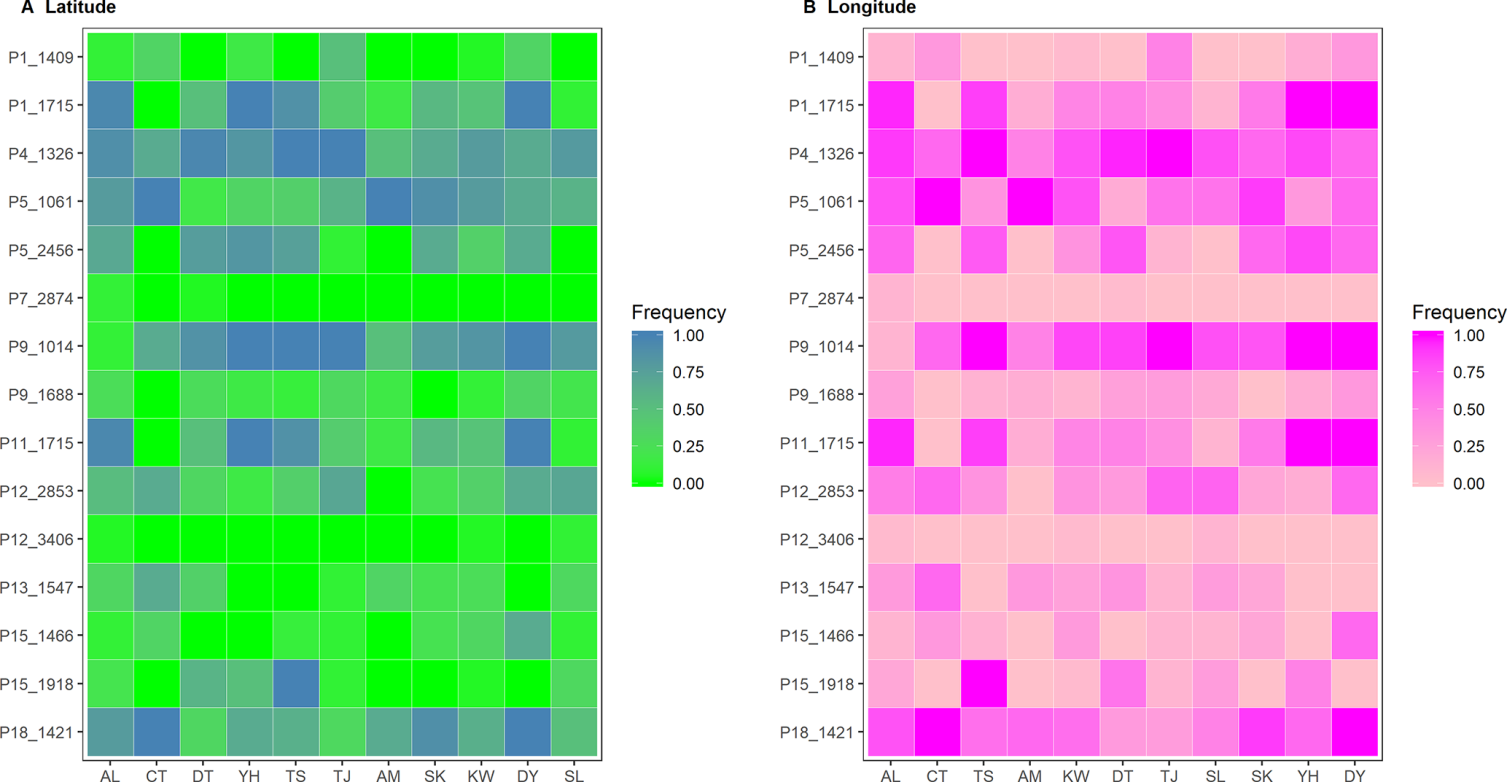
Heatmap of allele frequencies of the 15 outlier loci identified by either BAYESCAN or DFDIST. The sequence of populations was arranged according to **(A)** latitude or **(B)** longitude.

Six and nine nonoverlapped loci, respectively, were identified as *F*_ST_ outliers by BAYESCAN and DFDIST (**Table 4**). Allele frequencies of these outlier loci across populations arranged either latitudinally or longitudinally were depicted in **Figure 5**. Samβada and LFMM found significant associations of 18 and 83 loci, among the 482 loci scored, respectively, with at least one environmental variable. The Wald and G scores in the Samβada analysis were reported in **Supplementary Table 7**. The corresponding *Z*-score, −log_10_(*P* value), and adjusted *P* value of candidate outlier loci identified using LFMM were summarized in **Supplementary Table 8**. For the 15 loci identified either by BAYESCAN or DFDIST, Samβada found five (P1_1715, P5_2456, P9_1014, P11_1715, and P15_1918) and LFMM found seven (P1_1409, P1_1715, P5_2456, P11_1715, P12_2853, P13_1547, and P15_1446) loci, respectively, strongly correlated with environmental variables (**Table 4**). Of the 15 loci identified either by BAYESCAN or DFDIST, nine (P1_1715, P4_1326, P5_1061, P5_2456, P9_1014, P11_1715, P13_1547, P15_1918, and P18_1421) were found to be strongly associated with environmental variables using Bayesian logistic regression (the *stan_glm* function of R package rstanarm). In summary, 12 AFLP loci (2.49%) were identified either by BAYESCAN or DFDIST and associated strongly with environmental variables using Samβada, LFMM, and rstanarm. Results showed that annual mean temperature, annual precipitation, and slope were closely associated with a large proportion of the 12 *F*_ST_ outliers identified by BAYESCAN and DFDIST; and aspect, annual temperature range, NDVI, PET, and RainD associated strongly only with a minor proportion of these *F*_ST_ outliers. (**Table 4**).

**Table 4:** Potential outliers associated with environmental variables.

Markers	BAYESCAN log_10_PO	DFDIST	Samβada, LFMM, and rstanarm							
			Aspect	BIO1	BIO7	BIO12	NDVI	PET	RainD	Slope
P1_1409		0.0041					b			
P1_1715	0.6723			*,**		B		*,**		a,*
P4_1326		0.0075					*			*,**
P5_1061		0.0001		*,**		*				*,**
P5_2456	1.1684			b,*,**	a	a			*	a,*,**
P7_2874		0.0067								
P9_1014	1.4171			*,**		a,*,**	*			
P9_1688		0.0095								
P11_1715	0.6865			*,**		B		*,**		a,*
P12_2853		0.0098		B						
P12_3406		0.0080								
P13_1547		0.0017	B	*						
P15_1446	1.5961				a	a,B			a	a
P15_1918	0.8566					*				*,**
P18_1421		0.0089								*,**

Fifteen potential outliers were identified by F_ST_ genome scan methods (BAYESCAN and DFDIST) and 12 of them were found to be strongly associated with environmental variables using regression approach (Samβada, LFMM, and rstanarm).

a and b represent significant correlation of AFLP markers with individual environmental variables identified, respectively, by Samβada and LFMM. B represents a |Z| ≥ 1.5 in LFMM analysis.

*,** significance based on 95% and 99% posterior credible intervals for the potential outliers found to have strongly correlated with environmental variables using the stan_glm function of R package rstanarm.

Aspect (0–360°) and slope (0–90°).

BIO1, annual mean temperature; BIO7, annual temperature range; BIO12, annual precipitation; RainD, number of rainfall days per year. NDVI, normalized difference vegetation index; PET,The annual total potential evapotranspiration.

## Discussion

### Genetic Diversity

The average *uH*_E_ ± SD (= 0.252 ± 0.016) in the present study was found to be higher compared to that of the previous study examined for *C. konishii* (*uH*_E_ = 0.184; Chung et al., 2004). The higher number of amplification primer combinations used in the present study could be the cause of the discrepancy compared with the previous AFLP study, which may result in higher interlocus variance (Nei, 1987). Although there is no direct evidence for the relationship between the number of amplification primer pairs and the level of genetic diversity, higher interlocus variance was found to contribute more to the total variance associated with the level of genetic diversity compared to intralocus variance in *Pinus pinaster* (Ribeiro et al., 2002). Moreover, more amplified AFLP loci, though with higher error rate, could be positively correlated with higher level of genetic diversity (Zhang and Hare, 2012). Additionally, higher number of amplification primer pairs used could have amplified fragments spanning more chromosomal loci and contributed to the higher level of genetic diversity.

Based on the result of the present study, the average level of *C. konishii* genetic diversity appeared to be comparable to other conifers occurring in Taiwan that have been examined based on AFLP such as *Keteleeria davidiana* var. *formosana* (average *uH*_E_ = 0.233, Fang et al., 2013) and *T. cryptomerioides* (average *uH*_E_ = 0.236, Li et al., 2018). The average level of *C. konishii* genetic diversity was also found to be comparable to the average level of AFLP diversity (= 0.230) for 13 plant species in general summarized in Nybom (2004). Despite *C. konishii* covers only a small latitudinal range, it distributed across complex topography and habitat heterogeneity in mountain ranges (Su, 1984; Li et al., 2013) and hence could have contributed to the accumulation of genetic diversity (Hewitt, 1996; Petit et al., 2003; Hewitt, 2004).

### Mountain Ranges as a Physical Barrier to Genetic Exchange

The spatial distribution of genetic variability within natural plant populations can be shaped by population demographic history and geography through the combined effects of genetic drift, gene flow, and selection (Hoffmann and Sgrò, 2011). The discrepancy of the level of genetic differentiation based on AFLP was also observed as that of the level of genetic diversity between the present (**Table 3**) and the previous study (Chung et al., 2004). The *Φ*_ST_, *F*_ST_, and *θ* ^II^ estimates are consistent with the results of clustering analyses using LEA (**Figure 2**) and DAPC (**Figure 3**). In the present study, the level of population genetic differentiation was much smaller compared with the previous AFLP study based on AMOVA analysis (*Φ*_ST_ = 0.0827 vs. *Φ*_ST_ = 0.2460, respectively). However, the level of genetic differentiation of the present study is consistent with the levels of genetic differentiation found based on allozyme (*F*_ST_ = 0.029) (Lin et al., 1998) and ptDNA (*G*_ST_ = 0.073) (Hwang et al., 2003). The higher level of genetic differentiation among populations found in the previous study (Chung et al., 2004) could have arisen in part due to the smaller number of genomic regions amplified, compared with the present study, and harvested a relatively smaller fraction of ancestral polymorphisms contributing to the higher level of population differentiation. Nonetheless, the levels of population genetic structure estimated (Φ_ST_, *F*_ST_, and θ^II^) in the present study were consistent with the results found in other conifers occurring in Taiwan including *K. davidiana* var. *formosana* (Fang et al., 2013) and *T. cryptomerioides* (Li et al., 2018) based on AFLP. In addition, these results are in accordance with the general realization that conifers typically have high rates of effective pollen dispersal resulting in low population differentiation because of long distance wind pollination based on allozyme data (Hamrick et al., 1992; Hamrick and Godt, 1996).

Although low levels of across population and population pairwise *F*_ST_ were found (**Table 2**, **Supplementary Table 5**), genetic boundaries have been established by mountain ranges (**Figure 4**) and current gene flow among populations might have also been restricted by spatial environmental heterogeneity as suggested by PERMANOVA analysis (**Supplementary Table 6**). Additionally, Mantel test revealed no significant correlation of genetic distance matrix with geographic distance matrix suggesting no IBD pattern. These results suggest that the pattern of gene flow in *C. konishii* may not follow a stepping stone migration model (Kimura, 1953) due to the presence of physical dispersal barriers and sharp discontinuities in gene frequencies can be observed in populations in close proximity geographically (**Figures 1** and **5**). Our results suggest that the presence of physical barriers to gene flow and migration–drift equilibrium may not be reached, and a significant proportion of the observed low level of genetic differentiation reflects historical population expansion (Hwang et al., 2003) rather than current level of gene flow (Latta and Mitton, 1999; Turgeon and Bernatchez, 2001; Ringbauer et al., 2018). In the present study, the barrier effects of the HMR and the CMR and mountains in between of the HMR and the CMR could have played roles in shaping population differentiation of *C. konishii*. Moreover, mountains surrounding the AL population could have played a damping effect on dispersal between the AL and all other *C. konishii* populations, particularly between the AL and its nearby CT population, genetically (**Figure 4**). Therefore, natal dispersal within different geographic areas could have played an important role in shaping the genetic structure of *C. konishii*. However, environment could also be an important factor contributed to *C. konishii* population divergence because genetic variation is structured more by pure environment than by pure geography as revealed by the analysis of variation partitioning (**Table 3**). Our results suggest that environmental factors accompanied with physical separations have shaped the divergence between *C. konishii* populations. Ecotypes are likely to have arisen at local scales and the emergence of clusters of locally beneficial mutations forming genomic islands of divergence (Via, 2009; Wolf et al., 2010; Strasburg et al., 2012).

### The Most Important Environmental Variables for Adaptive Genetic Divergence in *C. konishii*

Current mating of *C. konishii* individuals among populations could be limited as suggested by significant population-level *I*_A_ and *r*D in 7 of the 11 populations examined, indicating nonrandom association of loci within these populations (**Table 1**). Local reduction in gene flow mediated by divergent selection and/or immigrant inviability that reduced survival and reproduction of immigrants might have also contributed to the detection of multilocus LD (Kawecki and Ebert, 2004; Nosil et al., 2005; Jump and Peñuelas, 2006). However, detection of LD does not ensure a lack of linkage equilibrium (Slatkin, 2008) and LD within populations may be related to the demographic history of species (Wall et al., 2002).

Our results based on analyses using BAYESCAN, DFDIST, Samβada, LFMM, and rstanarm found adaptive divergence of genetic variation (**Table 4**). Although BAYESCAN and DFDIST found no common outlier loci, regression approaches (Samβada, LFMM, and rstanarm) can be effective in testing their associations with environmental gradients (Stucki et al., 2017). Results showed that outlier AFLP loci were mainly driven by environmental variables including annual temperature range, annual precipitation, and slope (**Table 4**), and these environmental factors could have played major roles in driving outlier AFLP variation for local adaptation in *C. konishii*. Environmental variables including aspect, annual temperature range, NDVI, PET, and RainD could have played only minor roles as selective drivers in shaping adaptive divergence in *C. konishii*.

Strong correlations between environmental variables and population genetic variability can be provided as evidence for local adaptation and selection (Linhart and Grant, 1996). Temperature and precipitation have been found to be the two most important selective drivers for local adaptation in conifers (e.g., González-Martínez et al., 2006; Fang et al., 2013; Shih et al., 2018). Temporal and spatial variation in temperature and precipitation can influence fitness-related traits (Parmesan and Yohe, 2003; Hoffmann and Sgrò, 2011; Franks and Hoffmann, 2012) and consequently influence the survival of conifers (Brodribb et al., 2014). Topographic gradients over short distances in the rugged geographic landscape can play significant roles in shaping species composition of forest communities (Kitagawa et al., 2015). Topographic factors such as slope are important predictors of forest attributed to differences in radiation exposure and have a strong influence on the microclimate (Rosenberg et al., 1983; Bennie et al., 2008; Brousseau et al., 2015). Aspect and slope were found to be closely correlated with genetic variation within and between species (Nakazato et al., 2010; Monahan et al., 2012; Bothwell et al., 2013; Brousseau et al., 2015; Huang et al., 2015a; Huang et al., 2015b; Li et al., 2018). In the present study, slope could be the major topographic factor that played an important role in driving outlier AFLP variation for local adaptation (**Table 4**).

Environmental variation has been correlated with forest ecosystem properties in various geographic regions, suggesting the sensitivity of species composition responding to environmental conditions (Vandewalle et al., 2010; Roscher et al., 2012; Zhang et al., 2014). NDVI is a proxy to photosynthetic activity representing the level of vegetation greenness and has been shown to be correlated with intraspecific and interspecific adaptive divergence (Nakazato et al., 2010; Huang et al., 2015a; Huang et al., 2015b; Chen et al., 2017; Li et al., 2018). RainD represents the number of rainfall days per year, which was found to be associated with adaptive genetic variation of an angiosperm species, *Rhododendron oldhamii*, occur in Taiwan (Hsieh et al., 2013). PET was identified to be closely correlated with adaptive genetic variation between genetic lineages of a coniferous species, *T. cryptomerioides*, occurring in Taiwan, mainland China, and Vietnam (Li et al., 2018).

Our results suggest that ecologically relevant selective drivers involved in population adaptive divergence of *C. konishii*. Spatial environmental heterogeneity might have invoked genetic divergence among populations and in consequence the formation of local adaptation.

## Conclusions

The maintenance of genetic ecotypes adapting to varying environmental conditions can be critical for the conservation of species in the face of global climate change. Environmental variables might have exerted effects in shaping adaptive evolution of genetic variation. In the present study, we found annual mean temperature, annual precipitation, and slope could be the most important environmental factors that played crucial roles in shaping adaptive genetic divergence. Nonetheless, aspect, annual temperature range, NDVI, PET, and RainD, though might have played only minor roles, could still be important selective drivers of local adaptation in *C. konishii* populations. In addition, geographic barriers to gene flow exerted by mountain ranges could have played an important role in restricting adaptive genetic divergence at local scales. The three genetic clusters revealed by LEA and DAPC can be employed as evolutionarily significant units for the future conservation program of this species.

## Author Contributions

S-YH proposed, funded, and designed the research; Y-SL, K-MS, J-DC, and S-YH collected samples; Y-SL, K-MS, and C-TC performed research; S-YH, Y-SL, K-MS, and C-TC analyzed data; S-YH and Y-SL wrote the paper. All authors have read and approved the final manuscript.

## Funding

This work was funded by the Taiwan Ministry of Science and Technology under grant number of MOST 106-2313-B-003-001-MY3.

All plant materials collected conformed to the regulations of the Taiwan Ministry of Science and Technology.

## Conflict of Interest Statement

The authors declare that the research was conducted in the absence of any commercial or financial relationships that could be constructed as a potential conflict of interest.

## Supplementary Materials

Data Sheet 1 Comma-separated value file for scored AFLP markers of the 11 populations (n = 115) of Cunninghamia konishii. The first two columns recorded the information of sample ID and population code. The rest of columns represent presence/absence of markers.
