# Supplementary material for: Testing the Effect of Mountain Ranges as a Physical Barrier to Current Gene Flow and Environmentally Dependent Adaptive Divergence in Cunninghamia konishii (Cupressaceae)
Source: Front Genet. 2019 Aug 9;10:742. doi: 10.3389/fgene.2019.00742 (PMC6697026; doi:10.3389/fgene.2019.00742)
Supplement: Supplementary file 3 [file Table_1.docx]

**Supplementary Table 1.** Primer combinations, number of markers, and error rate per locus in AFLP technique

| Primer combination | Number of markers | Error rate (%) |
| --- | --- | --- |
|  |  |  |
| 1 E00 AAC + M00 CTCAT | 48 | 2.21 |
| 2 E00 CCC + M00 CTCAT | 35 | 2.02 |
| 3 E00 CCC + M00 CTCCA | 31 | 1.73 |
| 4 E00 TAA + M00 CTCCA | 35 | 1.44 |
| 5 E00 TAA + M00 CTCGG | 32 | 1.52 |
| 6 E00 AAC + M00 CTCGT | 45 | 1.60 |
| 7 E00 AAC + M00 CTGCT | 28 | 2.22 |
| 8 E00 TAA + M00 CTGCC | 36 | 2.07 |
| 9 E00 TAA + M00 CTGCT | 56 | 1.65 |
| 10 E00 AGC + M00 CTCGT | 28 | 2.33 |
| 11 E00 CAC + M00 CTCGT | 40 | 1.77 |
| 12 E00 CCC + M00 CTCGT | 34 | 2.00 |
| 13 E00 CCC + M00 CTCTC | 34 | 2.20 |
| Total (Average) | 482 (37.1) | (1.91) |

E00 (5’-GACTGCGTACCAATTC-3’)

M00 (5’-GATGAGTCCTGAGTAA-3’)
