# Supplementary material for: Testing the Effect of Mountain Ranges as a Physical Barrier to Current Gene Flow and Environmentally Dependent Adaptive Divergence in Cunninghamia konishii (Cupressaceae)
Source: Front Genet. 2019 Aug 9;10:742. doi: 10.3389/fgene.2019.00742 (PMC6697026; doi:10.3389/fgene.2019.00742)
Supplement: Supplementary file 4 [file Table_2.docx]

**Supplementary Table 2.** Site environmental variables of the eight populations of *Cunninghamia konishii*. See **Table 1** for abbreviations of the eight populations of *C. konishii*.

|  | Environmental variables | | | | | | | |
| --- | --- | --- | --- | --- | --- | --- | --- | --- |
| Population | Aspect | BIO1 | BIO7 | BIO12 | NDVI | PET | RainD | Slope |
| AL | 112.7 | 133 | 159 | 3797 | 0.80 | 1317.1 | 12.2 | 54.0 |
| AM | 66.3 | 111 | 166 | 3140 | 0.85 | 1444.9 | 10.6 | 4.9 |
| CT | 346.0 | 165 | 160 | 2537 | 0.79 | 725.7 | 12.0 | 10.2 |
| DT | 212.1 | 115 | 159 | 3469 | 0.80 | 1648.2 | 12.7 | 42.9 |
| DY | 91.8 | 157 | 168 | 2861 | 0.81 | 304.7 | 14.9 | 30.2 |
| KW | 137.2 | 117 | 169 | 2748 | 0.87 | 1003.0 | 10.7 | 19.3 |
| SK | 234.1 | 118 | 166 | 2832 | 0.84 | 1413.1 | 12.2 | 26.6 |
| SL | 130.2 | 152 | 174 | 2413 | 0.88 | 613.9 | 11.6 | 32.4 |
| TJ | 112.1 | 140 | 165 | 2457 | 0.72 | 1606.5 | 11.5 | 33.9 |
| TS | 201.4 | 115 | 167 | 3130 | 0.85 | 1068.3 | 10.6 | 37.7 |
| YH | 142.6 | 130 | 160 | 2747 | 0.85 | 1011.6 | 12.8 | 43.1 |

*Aspect (0–360°) and slope (0–90°).*

*BIO1, Annual mean temperature; BIO7, annual temperature range; BIO12, annual precipitation; NDVI, normalized difference vegetation index; PET, annual total potential evapotranspiration; RainD, number of rainfall days per year.*
