# Supplementary material for: Testing the Effect of Mountain Ranges as a Physical Barrier to Current Gene Flow and Environmentally Dependent Adaptive Divergence in Cunninghamia konishii (Cupressaceae)
Source: Front Genet. 2019 Aug 9;10:742. doi: 10.3389/fgene.2019.00742 (PMC6697026; doi:10.3389/fgene.2019.00742)
Supplement: Supplementary file 5 [file Table_3.docx]

**Supplementary Table 3.** Summary of Tukey’s post-hoc pairwise population comparisons of the mean unbiased expected heterozygosity using linear mixed effect model. In linear mixed effect model, populations were treated as fixed effect and locus as a random effect.

| Population | Difference | *Z* | *P* |
| --- | --- | --- | --- |
| AL - AM | 0.048469 | 4.045 | 0.0026 |
| AL - CT | 0.013612 | 1.136 | 0.9885 |
| AL - DT | 0.027866 | 2.326 | 0.416 |
| AL - DY | 0.019144 | 1.598 | 0.8837 |
| AL - KW | -0.00405 | -0.338 | 1 |
| AL - SK | 0.001971 | 0.165 | 1 |
| AL - SL | 0.013289 | 1.109 | 0.9905 |
| AL - TJ | 0.024677 | 2.06 | 0.6065 |
| AL - TS | 0.038954 | 3.251 | 0.0452 |
| AL - YH | 0.038095 | 3.18 | 0.0563 |
| AM - CT | -0.03486 | -2.909 | 0.1199 |
| AM - DT | -0.0206 | -1.72 | 0.8258 |
| AM - DY | -0.02932 | -2.447 | 0.3362 |
| AM - KW | -0.05252 | -4.384 | 0.0006 |
| AM - SK | -0.0465 | -3.881 | 0.005 |
| AM - SL | -0.03518 | -2.936 | 0.1117 |
| AM - TJ | -0.02379 | -1.986 | 0.6591 |
| AM - TS | -0.00951 | -0.794 | 0.9994 |
| AM - YH | -0.01037 | -0.866 | 0.9988 |
| CT - DT | 0.014254 | 1.19 | 0.9837 |
| CT - DY | 0.005533 | 0.462 | 1 |
| CT - KW | -0.01766 | -1.474 | 0.9285 |
| CT - SK | -0.01164 | -0.972 | 0.9967 |
| CT - SL | -0.00032 | -0.027 | 1 |
| CT - TJ | 0.011065 | 0.924 | 0.9978 |
| CT - TS | 0.025342 | 2.115 | 0.5663 |
| CT - YH | 0.024484 | 2.043 | 0.6181 |
| DT - DY | -0.00872 | -0.728 | 0.9997 |
| DT - KW | -0.03192 | -2.664 | 0.2164 |
| DT - SK | -0.02589 | -2.161 | 0.5328 |
| DT - SL | -0.01458 | -1.217 | 0.9808 |
| DT - TJ | -0.00319 | -0.266 | 1 |
| DT - TS | 0.011088 | 0.925 | 0.9978 |
| DT - YH | 0.010229 | 0.854 | 0.9989 |
| DY - KW | -0.0232 | -1.936 | 0.6934 |
| DY - SK | -0.01717 | -1.433 | 0.9402 |
| DY - SL | -0.00586 | -0.489 | 1 |
| DY - TJ | 0.005533 | 0.462 | 1 |
| DY - TS | 0.01981 | 1.653 | 0.859 |
| DY - YH | 0.018951 | 1.582 | 0.8904 |
| KW - SK | 0.006024 | 0.503 | 1 |
| KW - SL | 0.017342 | 1.447 | 0.9364 |
| KW - TJ | 0.028729 | 2.398 | 0.3678 |
| KW - TS | 0.043007 | 3.589 | 0.0147 |
| KW - YH | 0.042148 | 3.518 | 0.0189 |
| SK - SL | 0.011318 | 0.945 | 0.9974 |
| SK - TJ | 0.022706 | 1.895 | 0.7208 |
| SK - TS | 0.036983 | 3.087 | 0.0739 |
| SK - YH | 0.036124 | 3.015 | 0.0903 |
| SL - TJ | 0.011388 | 0.95 | 0.9973 |
| SL - TS | 0.025665 | 2.142 | 0.5467 |
| SL - YH | 0.024806 | 2.07 | 0.5987 |
| TJ - TS | 0.014277 | 1.192 | 0.9835 |
| TJ - YH | 0.013418 | 1.12 | 0.9897 |
| TS - YH | -0.00086 | -0.072 | 1 |
