# Supplementary material for: Testing the Effect of Mountain Ranges as a Physical Barrier to Current Gene Flow and Environmentally Dependent Adaptive Divergence in Cunninghamia konishii (Cupressaceae)
Source: Front Genet. 2019 Aug 9;10:742. doi: 10.3389/fgene.2019.00742 (PMC6697026; doi:10.3389/fgene.2019.00742)
Supplement: Supplementary file 6 [file Table_4.docx]

**Supplementary Table 4.** The levels of differentiation and inbreeding estimated using HICKORY based on genetic markers of *Cunninghamia konishii* and the best fitting model is in bold.

| Model | *θ^II^* (95% CI) | *f* (95% CI) | $\overline{D}$ | $\hat{D}$ | *pD* | DIC |
| --- | --- | --- | --- | --- | --- | --- |
| ***Full*** | **0.0478 (0.040 – 0.066)** | **0.1374 (0.005 – 0.927)** | **11919.2** | **10336.0** | **1583.22** | **13502.5** |
| *f* = 0 | 0.0440 (0.039 – 0.049) |  | 11908.4 | 10260.3 | 1648.12 | 13556.5 |
| *θ* = 0 |  | 0.9907 (0.966 – 1.000) | 14909.3 | 14478.6 | 430.624 | 15339.9 |
| *f*-free | 0.0848 (0.071 – 0.101) | 0.4969 (0.022 – 0.976) | 11840.4 | 9753.52 | 2086.83 | 13927.2 |

*θ^II^, is the best Bayesian inference estimate of the proportion of genetic diversity due to differences among populations, and is an analogue to F_ST_.*

*D̅, is a measure of how well the model fits the data (smaller values indicate a better fit).*

$\hat{D}$*, is a measure of how well the best point estimate fits the data.*

*pD, is a measure of model complexity, i.e., the effective number of parameters being estimated (pD = D̅-*$\hat{D}$*).*

*DIC, deviance information criterion.*

*f, an estimate of F_IS_, inbreeding within a population*
