# Supplementary material for: Testing the Effect of Mountain Ranges as a Physical Barrier to Current Gene Flow and Environmentally Dependent Adaptive Divergence in Cunninghamia konishii (Cupressaceae)
Source: Front Genet. 2019 Aug 9;10:742. doi: 10.3389/fgene.2019.00742 (PMC6697026; doi:10.3389/fgene.2019.00742)
Supplement: Supplementary file 7 [file Table_5.docx]

**Supplementary Table 5.** Pairwise *F*_ST_ between populations of *Cunninghamia* *konishii* using ARLEQUIN with 10,000 permutations.

|  | Pairwise *F*_ST_ (bottom diagonal) and *P* (upper diagonal) values | | | | | | | | | | |
| --- | --- | --- | --- | --- | --- | --- | --- | --- | --- | --- | --- |
|  | AL | AM | CT | DT | DY | KW | SK | SL | TJ | TS | YH |
| AL |  | 0 | 0.00059 | 0 | 0.00149 | 0 | 0.00069 | 0 | 0 | 0 | 0 |
| AM | 0.15427 |  | 0.29403 | 0 | 0.01139 | 0.00644 | 0.01178 | 0.00267 | 0.0003 | 0.0001 | 0.00119 |
| CT | 0.14214 | 0.01399 |  | 0.00099 | 0.09682 | 0.03168 | 0.06287 | 0.01307 | 0.01643 | 0.00634 | 0.01247 |
| DT | 0.10015 | 0.12383 | 0.15678 |  | 0.00059 | 0.0001 | 0 | 0 | 0 | 0.00396 | 0.29175 |
| DY | 0.11593 | 0.31435 | 0.30325 | 0.20347 |  | 0.01802 | 0.06182 | 0.00297 | 0.00386 | 0.00574 | 0.01208 |
| KW | 0.06873 | 0.07147 | 0.07467 | 0.04878 | 0.09347 |  | 0.4658 | 0.05712 | 0.05881 | 0.0069 | 0.0195 |
| SK | 0.05178 | 0.09707 | 0.09708 | 0.0624 | 0.0856 | 0 |  | 0.00178 | 0.00178 | 0.0004 | 0.01317 |
| SL | 0.13409 | 0.05759 | 0.08875 | 0.07447 | 0.1901 | 0.02104 | 0.05817 |  | 0.50025 | 0 | 0 |
| TJ | 0.11918 | 0.08719 | 0.09346 | 0.06646 | 0.18134 | 0.01995 | 0.05697 | 0 |  | 0.0001 | 0.0004 |
| TS | 0.12557 | 0.18775 | 0.22607 | 0.04318 | 0.235 | 0.07742 | 0.08425 | 0.11383 | 0.10316 |  | 0.0123 |
| YH | 0.08101 | 0.14165 | 0.17223 | 0.00638 | 0.2041 | 0.04653 | 0.05441 | 0.10218 | 0.09509 | 0.04425 |  |
