# Supplementary material for: Testing the Effect of Mountain Ranges as a Physical Barrier to Current Gene Flow and Environmentally Dependent Adaptive Divergence in Cunninghamia konishii (Cupressaceae)
Source: Front Genet. 2019 Aug 9;10:742. doi: 10.3389/fgene.2019.00742 (PMC6697026; doi:10.3389/fgene.2019.00742)
Supplement: Supplementary file 8 [file Table_6.docx]

**Supplementary Table 6.** *P* values of pairwise group and population comparisons of the eight environmental variables using PERMANOVA.

|  | Cluster 1 | Cluster 2 |  |  |  |  |  |  |  |  |
| --- | --- | --- | --- | --- | --- | --- | --- | --- | --- | --- |
| Cluster 2 | 0.047 |  |  |  |  |  |  |  |  |  |
| Cluster 3 | 0.0015 | 0.0015 |  |  |  |  |  |  |  |  |
|  |  |  |  |  |  |  |  |  |  |  |
|  | AL | AM | CT | DT | DY | KW | SK | SL | TJ | TS |
| AM | 0.0026 |  |  |  |  |  |  |  |  |  |
| CT | 1 | 1 |  |  |  |  |  |  |  |  |
| DT | 1 | 1 | 0.0029 |  |  |  |  |  |  |  |
| DY | 1 | 1 | 1 | 1 |  |  |  |  |  |  |
| KW | 1 | 1 | 1 | 0.0026 | 1 |  |  |  |  |  |
| SK | 1 | 1 | 1 | 1 | 1 | 0.0026 |  |  |  |  |
| SL | 0.0026 | 0.0026 | 0.0069 | 1 | 0.0026 | 1 | 0.0026 |  |  |  |
| TJ | 1 | 0.0026 | 0.0085 | 1 | 0.0085 | 1 | 0.0026 | 1 |  |  |
| TS | 0.0026 | 0.0026 | 0.0048 | 0.0026 | 0.0122 | 0.0026 | 0.0026 | 0.0026 | 0.0026 |  |
| YH | 1 | 1 | 0.0177 | 0.0026 | 0.0341 | 0.0026 | 0.0048 | 0.0026 | 0.0026 | 1 |

*Significance determined by 999 permutations and a false discovery rate of 5%.*
