# Supplementary material for: Testing the Effect of Mountain Ranges as a Physical Barrier to Current Gene Flow and Environmentally Dependent Adaptive Divergence in Cunninghamia konishii (Cupressaceae)
Source: Front Genet. 2019 Aug 9;10:742. doi: 10.3389/fgene.2019.00742 (PMC6697026; doi:10.3389/fgene.2019.00742)
Supplement: Supplementary file 9 [file Table_7.docx]

**Supplementary Table 7.** Eighteen candidate loci significantly associated with environmental variables identified by the Samβada approach.

| Locus | Environmental variables | Adjusted P-value of G score | Adjusted P-value of Wald score |
| --- | --- | --- | --- |
| P1_1715 | Slope | 3.1603E-03 | 1.7475E-02 |
| P1_2126 | BIO7 | 1.1533E-02 | 3.6426E-02 |
| P4_1153 | BIO12 | 3.7450E-03 | 1.7475E-02 |
| P4_2337 | BIO12 | 9.9939E-04 | 1.7475E-02 |
|  | Slope | 2.3701E-03 | 1.7475E-02 |
| P5_2456 | BIO7 | 3.2989E-03 | 1.7475E-02 |
|  | BIO12 | 2.0256E-03 | 1.2136E-02 |
|  | Slope | 3.1603E-03 | 1.7598E-02 |
| P6_1346 | Slope | 2.7090E-03 | 3.6426E-02 |
| P6_1981 | BIO12 | 3.7082E-04 | 7.6429E-03 |
| P9_1014 | BIO12 | 4.4494E-04 | 5.9373E-03 |
| P9_1804 | Slope | 7.7880E-04 | 1.7475E-02 |
| P9_2084 | Slope | 1.9829E-03 | 1.7475E-02 |
| P11_1715 | Slope | 3.1603E-03 | 1.7475E-02 |
| P12_1658 | Slope | 2.7090E-03 | 2.0497E-02 |
| P13_1040 | BIO7 | 3.7082E-04 | 5.9373E-03 |
|  | BIO12 | 7.7097E-04 | 6.4297E-03 |
|  | Slope | 9.3220E-03 | 3.8880E-02 |
| P15_1446 | BIO7 | 4.5153E-04 | 5.9373E-03 |
|  | BIO12 | 4.4494E-04 | 5.9373E-03 |
|  | RainD | 3.7082E-04 | 5.9373E-03 |
|  | Slope | 6.6185E-04 | 8.2474E-03 |
| P15_1739 | Slope | 1.0505E-02 | 4.2651E-02 |
| P15_1776 | Slope | 3.8251E-03 | 2.0468E-02 |
| P17_1783 | Slope | 6.9986E-03 | 2.7992E-02 |
| P17_2385 | BIO7 | 3.8413E-03 | 1.7475E-02 |
|  | Slope | 3.7082E-04 | 5.9373E-03 |
|  | BIO12 | 1.0110E-03 | 1.2136E-02 |

*Aspect (0–360°) and slope (0–90°).*

*BIO1, Annual mean temperature; BIO7, annual temperature range; BIO12, annual precipitation; NDVI, normalized difference vegetation index, PET, annual total potential evapotranspiration; RainD, number of rainfall days per year.*
