# Supplementary material for: Testing the Effect of Mountain Ranges as a Physical Barrier to Current Gene Flow and Environmentally Dependent Adaptive Divergence in Cunninghamia konishii (Cupressaceae)
Source: Front Genet. 2019 Aug 9;10:742. doi: 10.3389/fgene.2019.00742 (PMC6697026; doi:10.3389/fgene.2019.00742)
Supplement: Supplementary file 10 [file Table_8.docx]

**Supplementary Table 8.** Eighty-three candidate loci significantly associated with environmental variables identified by the latent factor mixed model (LFMM) approach.

| Locus | Environmental variables | *Z*-score | -log_10_(*P*-value) | adjusted *P*-value |
| --- | --- | --- | --- | --- |
| AFLP |  |  |  |  |
| P1_1042 | Aspect | 1.42193 | 4.74262 | 1.8087E-05 |
| P1_1143 | BIO1 | -1.52771 | 4.35201 | 4.4462E-05 |
| P1_1388 | BIO7 | -1.70883 | 4.71295 | 1.9366E-05 |
| P1_1409 | NDVI | -1.44864 | 4.53891 | 2.8913E-05 |
| P1_1715 | BIO12 | 1.56942 | 4.29471 | 5.0733E-05 |
| P1_2126 | Aspect | 1.29514 | 4.02582 | 9.4228E-05 |
|  | BIO7 | -2.87227 | 12.15884 | 6.9368E-13 |
|  | BIO12 | 2.15692 | 7.59177 | 2.5599E-08 |
|  | NDVI | -1.68780 | 5.95731 | 1.1033E-06 |
| P1_2284 | Aspect | 1.51135 | 5.28470 | 5.1916E-06 |
| P1_3187 | Aspect | 1.63708 | 6.09828 | 7.9748E-07 |
| P3_1116 | Aspect | 1.19732 | 3.51409 | 3.0613E-04 |
| P3_1449 | Aspect | -1.97001 | 8.54460 | 2.8536E-09 |
| P3_1652 | BIO1 | 1.73261 | 5.43816 | 3.6462E-06 |
| P3_1740 | BIO12 | 2.08052 | 7.10835 | 7.7920E-08 |
| P3_1767 | Aspect | -1.45690 | 4.95101 | 1.1194E-05 |
|  | NDVI | -1.93292 | 7.61850 | 2.4071E-08 |
| P3_1922 | NDVI | -1.57184 | 5.24472 | 5.6922E-06 |
|  | PET | 2.31696 | 6.10672 | 7.8213E-07 |
| P3_2404 | BIO1 | 1.42778 | 3.86746 | 1.3569E-04 |
|  | BIO12 | -1.82101 | 5.58864 | 2.5784E-06 |
| P3_2539 | BIO1 | -1.40244 | 3.74928 | 1.7812E-04 |
| P3_3102 | BIO7 | 1.55631 | 4.00033 | 9.9925E-05 |
|  | NDVI | 1.59288 | 5.37053 | 4.2606E-06 |
| P4_1153 | BIO12 | -2.25223 | 8.21789 | 6.0550E-09 |
| P4_2337 | BIO12 | -1.84163 | 5.70250 | 1.9838E-06 |
| P5_1088 | Aspect | 1.15621 | 3.30971 | 4.9011E-04 |
| P5_1206 | Slope | -2.13946 | 4.27797 | 5.2727E-05 |
| P5_1285 | Aspect | -1.51646 | 5.31659 | 4.8240E-06 |
| P5_1540 | BIO1 | 1.65385 | 5.00588 | 9.8655E-06 |
| P5_1638 | BIO12 | -1.49857 | 3.96197 | 1.0915E-04 |
| P5_1832 | Aspect | 1.13676 | 3.21520 | 6.0926E-04 |
| P5_2263 | Aspect | -1.38823 | 4.54618 | 2.8433E-05 |
| P5_2456 | BIO1 | -1.38587 | 3.67302 | 2.1232E-04 |
| P5_2882 | BIO7 | 1.74574 | 4.89449 | 1.2750E-05 |
| P6_1216 | BIO12 | 1.44957 | 3.73993 | 1.8200E-04 |
| P6_1308 | BIO1 | -1.53086 | 4.36776 | 4.2879E-05 |
| P6_1346 | BIO12 | -2.00745 | 6.66135 | 2.1810E-07 |
|  | NDVI | 1.61058 | 5.47757 | 3.3299E-06 |
|  | Slope | -2.82743 | 7.03977 | 9.1249E-08 |
| P6_1653 | Aspect | 1.28672 | 3.98036 | 1.0463E-04 |
| P6_1981 | BIO12 | 1.39044 | 3.48076 | 3.3055E-04 |
| P6_2055 | BIO7 | -1.77501 | 5.04096 | 9.0999E-06 |
|  | NDVI | -2.03775 | 8.39344 | 4.0417E-09 |
| P7_1739 | Aspect | 1.54752 | 5.51258 | 3.0720E-06 |
| P7_1865 | Aspect | 1.29298 | 4.01413 | 9.6798E-05 |
|  | BIO7 | -1.67074 | 4.52933 | 2.9557E-05 |
| P7_1908 | Aspect | 1.48086 | 5.09646 | 8.0083E-06 |
| P7_1975 | BIO1 | 1.46619 | 4.05022 | 8.9080E-05 |
| P7_2088 | NDVI | -1.75426 | 6.38689 | 4.1031E-07 |
| P7_2410 | NDVI | 1.27497 | 3.63310 | 2.3276E-04 |
| P7_2524 | NDVI | -1.32470 | 3.88186 | 1.3126E-04 |
| P7_2541 | Aspect | 1.35382 | 4.35003 | 4.4665E-05 |
| P7_2629 | Aspect | 1.43510 | 4.82056 | 1.5116E-05 |
| P7_2781 | Aspect | 1.59274 | 5.80449 | 1.5686E-06 |
| P9_1130 | BIO1 | 1.43148 | 3.88488 | 1.3035E-04 |
| P9_1395 | BIO7 | 1.55604 | 3.99912 | 1.0020E-04 |
| P9_1611 | Aspect | 1.41340 | 4.69250 | 2.0300E-05 |
| P9_2018 | Aspect | 1.35045 | 4.33106 | 4.6659E-05 |
|  | Slope | 2.26224 | 4.71911 | 1.9094E-05 |
| P11_1042 | Aspect | 1.48727 | 5.13574 | 7.3158E-06 |
| P11_1143 | BIO1 | -1.57186 | 4.57549 | 2.6577E-05 |
| P11_1283 | Aspect | -1.33626 | 4.25165 | 5.6020E-05 |
| P11_1388 | BIO7 | -1.87136 | 5.53892 | 2.8912E-06 |
| P11_1409 | NDVI | -1.47961 | 4.71138 | 1.9437E-05 |
| P11_1715 | BIO12 | 1.56807 | 4.28824 | 5.1494E-05 |
| P11_2032 | BIO1 | -1.65082 | 4.98962 | 1.0242E-05 |
| P11_3187 | Aspect | 1.68139 | 6.39936 | 3.9869E-07 |
| P12_1092 | Aspect | -1.18302 | 3.44228 | 3.6117E-04 |
| P12_1189 | Aspect | -1.85329 | 7.63854 | 2.2986E-08 |
| P12_1931 | BIO12 | 1.92750 | 6.18943 | 6.4650E-07 |
| P12_2450 | BIO1 | 1.41866 | 3.82471 | 1.4972E-04 |
| P12_2529 | Aspect | 1.44737 | 4.89376 | 1.2771E-05 |
| P12_2853 | BIO1 | 1.64817 | 4.97542 | 1.0582E-05 |
| P12_4224 | Aspect | -1.14339 | 3.24726 | 5.6590E-04 |
|  | BIO1 | 1.66850 | 5.08489 | 8.2245E-06 |
| P13_1040 | Slope | 2.49861 | 5.63149 | 2.3362E-06 |
| P13_1547 | Aspect | 1.50619 | 5.25259 | 5.5899E-06 |
| P13_1675 | Aspect | 2.21363 | 10.60496 | 2.4834E-11 |
| P13_1940 | Aspect | 1.34940 | 4.32516 | 4.7298E-05 |
| P13_2090 | NDVI | -1.30083 | 3.76140 | 1.7322E-04 |
| P13_2301 | Aspect | 1.80515 | 7.28009 | 5.2470E-08 |
| P13_2345 | Aspect | 1.79178 | 7.18212 | 6.5748E-08 |
| P15_1202 | BIO7 | 1.55055 | 3.97459 | 1.0602E-04 |
| P15_1225 | BIO12 | -1.89747 | 6.01680 | 9.6205E-07 |
| P15_1338 | BIO12 | -1.53986 | 4.15420 | 7.0113E-05 |
| P15_1405 | Aspect | 1.12455 | 3.15659 | 6.9728E-04 |
| P15_1446 | BIO12 | -1.61689 | 4.52541 | 2.9826E-05 |
| P15_1556 | Aspect | 1.30106 | 4.05794 | 8.7510E-05 |
| P15_1676 | BIO12 | -2.13528 | 7.45318 | 3.5223E-08 |
| P15_1739 | PET | -2.10628 | 5.14861 | 7.1021E-06 |
| P15_1964 | Aspect | 1.76132 | 6.96147 | 1.0928E-07 |
| P17_1371 | BIO7 | 1.92169 | 5.80868 | 1.5535E-06 |
| P17_1705 | Aspect | 1.19643 | 3.50960 | 3.0931E-04 |
| P17_2244 | BIO12 | -1.65695 | 4.72495 | 1.8839E-05 |
| P18_1104 | Aspect | 1.58604 | 5.76075 | 1.7348E-06 |

*Aspect (0–360°) and slope (0–90°).*

*BIO1, Annual mean temperature; BIO7, annual temperature range; BIO12, annual precipitation; NDVI, normalized difference vegetation index, PET, annual total potential evapotranspiration; RainD, number of rainfall days per year.*
